# Transcriptome-Stable Isotope Probing Provides Targeted Functional and Taxonomic Insights Into Microaerobic Pollutant-Degrading Aquifer Microbiota

**DOI:** 10.3389/fmicb.2018.02696

**Published:** 2018-11-13

**Authors:** Lauren M. Bradford, Gisle Vestergaard, András Táncsics, Baoli Zhu, Michael Schloter, Tillmann Lueders

**Affiliations:** ^1^Institute of Groundwater Ecology, Helmholtz Zentrum München, Neuherberg, Germany; ^2^Section of Microbiology, University of Copenhagen, Copenhagen, Denmark; ^3^Research Unit Comparative Microbiome Analysis, Helmholtz Zentrum München, Neuherberg, Germany; ^4^Regional University Center of Excellence in Environmental Industry, Szent István University, Gödöllö, Hungary

**Keywords:** RNA-seq, RNA-SIP, metatranscriptomics, hydrocarbon degradation, groundwater, dioxygenases

## Abstract

While most studies using RNA-stable isotope probing (SIP) to date have focused on ribosomal RNA, the detection of ^13^C-labeled mRNA has rarely been demonstrated. This approach could alleviate some of the major caveats of current non-target environmental “omics.” Here, we demonstrate the feasibility of total RNA-SIP in an experiment where hydrocarbon-degrading microbes from a BTEX-contaminated aquifer were studied in microcosms with ^13^C-labeled toluene under microoxic conditions. From the total sequencing reads (∼30 mio. reads per density-resolved RNA fraction), an average of 1.2% of reads per sample were identified as non-rRNA, including mRNA. Members of the *Rhodocyclaceae* (including those related to *Quatrionicoccus* spp.) were most abundant and enriched in ^13^C-rRNA, while well-known aerobic degraders such as *Pseudomonas* spp. remained unlabeled. Transcripts related to cell motility, secondary metabolite formation and xenobiotics degradation were highly labeled with ^13^C. mRNA of phenol hydroxylase genes were highly labeled and abundant, while other transcripts of toluene-activation were not detected. Clear labeling of catechol 2,3-dioxygenase transcripts supported previous findings that some of these extradiol dioxygenases were adapted to low oxygen concentrations. We introduce a novel combination of total RNA-SIP with calculation of transcript-specific enrichment factors (EFs) in ^13^C-RNA, enabling a targeted approach to process-relevant gene expression in complex microbiomes.

## Introduction

Unraveling the activity of microorganisms in complex environments is a difficult but essential step toward understanding their metabolism and community interactions in processes like pollutant degradation. Stable isotope probing (SIP) of nucleic acids is widely applied to discover which organisms metabolize a particular substrate, and can provide insights into catabolic pathways used (Coyotzi et al., 2016; Lueders et al., 2016). As microbes metabolize an isotopically labeled substrate, the isotope is incorporated into their biomolecules, including nucleic acids. These can then be extracted and separated into labeled and unlabeled fractions by isopycnic centrifugation (Meselson and Stahl, 1958). Compared to DNA-SIP where DNA labeling only occurs during cell replication, RNA-SIP allows for a more dynamic perspective of specifically active populations (Manefield et al., 2002; Lueders et al., 2016).

The majority of RNA-SIP studies to this point have targeted small subunit ribosomal RNA (SSU rRNA) to taxonomically identify microbes involved in substrate metabolism. However, isopycnic gradients have the capacity to resolve both labeled rRNA and mRNA (Rickwood, 1992). Still, only a small number of studies have actually targeted functional transcripts from SIP gradients, and in most cases researchers looked at the transcripts using reverse-transcription PCR and fingerprinting (Huang et al., 2009; Pratscher et al., 2011; El Zahar Haichar et al., 2012). These methods can only provide insights into the labeling of very specific transcript pools and may suffer from primer bias or even a total lack of suitable PCR targets. Only two studies to date have combined mRNA-SIP with next generation sequencing. Dumont et al. (2013) studied the cycling of ^13^CH_4_ in oxic lake sediment cultures. Their workflow involved both rRNA depletion prior to isopycnic centrifugation and linear amplification of the mRNA recovered from gradient fractions to produce sufficient material for 454 sequencing. With the increased sequencing depth of Illumina platforms, it is now possible to directly investigate total RNA from heavy and light gradient fractions instead, recovering both taxonomic and functional information. This was first accomplished by Fortunato and Huber (2016), who used total RNA-SIP to study chemolithoautotrophic communities from a deep-sea hydrothermal vent in hydrogen-enriched incubations. The authors also discussed methodological caveats of sequencing gradient fractions with very little RNA. Total RNA sequencing (RNA-seq) can avoid many of the pitfalls of PCR and allows the discovery of transcripts in an undirected manner. However, since the total amount of RNA which can be loaded into a SIP gradient is typically limited to <1 μg (Whiteley et al., 2007), the low yield of RNA after fractionation represents a challenge for downstream analyses. Post-gradient linear amplification of RNA (Dumont et al., 2013) may provide means of increasing template amounts for sequencing, and has also been hypothesized to increase the proportion of mRNA in total RNA (Cao et al., 2010). Thus, linear amplification could be a viable strategy for improving total mRNA reads from gradient RNA.

Nucleic acid-based SIP has been widely applied to identify the organisms responsible for degrading environmental pollutants (Lueders et al., 2016). Though both aerobic and anaerobic hydrocarbon degraders have been intensively studied, degraders that thrive under microoxic conditions have received comparably little attention. Still, such degraders could be very relevant in habitats with fluctuating oxygen availability, or at redox gradients. They could differ from strictly aerobic or anaerobic degraders by more efficient terminal oxidases (Morris and Schmidt, 2013) and by a flexible use of oxygenase-based catabolic pathways and aerobic or anaerobic respiration (Kukor and Olsen, 1996; Wilson and Bouwer, 1997). Here, we aimed to contribute to this discussion by demonstrating that transcriptome-SIP can be applied to microaerobic degraders. Sediments from a long-term hydrocarbon-contaminated aquifer in Siklós, Hungary have been recently subjected to DNA-SIP with ^13^C-toluene in a microcosm study to investigate the microbes involved in biodegradation under microoxic conditions (Táncsics et al., 2018). Amplicon sequencing of 16S rRNA genes identified various lineages within the *Rhodocyclaceae* as the main toluene degraders, and subfamily 1.2.C extradiol dioxygenase genes affiliated to *Zoolgoea* spp. were found in ^13^C-labeled DNA. For the present study, we extracted highly ^13^C-labeled total RNA from the same microcosms after 7 days of microoxic incubation, to enable cross-comparison between amplicon-based DNA-SIP and our transcriptome-SIP method. Our results provide a proof-of-principle that transcriptome-SIP can actually be applied to pollutant-degrading complex microbiomes and that it can provide additional insights, beyond taxonomic identification, into the activity of uncultured degrader populations. We also test whether post-centrifugation linear amplification of gradient RNA offers a viable means of improving mRNA ratios in sequencing results.

## Materials and Methods

### Study Site and SIP Incubation

Details about the study site in Siklós, Hungary, sediment sampling, and the setup and incubation of microoxic SIP microcosms with ^13^C-labeled toluene can be found elsewhere (Táncsics et al., 2012, 2018). Briefly, sediment samples were taken from a monitoring well 6 m below ground surface in the center of a BTEX plume. Each microcosm consisted of a 100 ml serum bottle with 5 g (wet weight) sediment material, 50 ml artificial groundwater medium (Winderl et al., 2010), with a N_2_/CO_2_ (80/20, v/v) headspace. This amount of sediment was chosen to prevent redox stratification during incubation. Five microliter of non-labeled (^12^C) or fully labeled (^13^C_7_) toluene were added by injection with a glass syringe after headspace exchange. Abiotic control microcosms were set up in the same manner, but autoclaved 3x before addition of non-labeled toluene. These microcosms controlled for possible abiotic toluene loss during incubation (Táncsics et al., 2018). Microcosms were incubated at 16°C with 145 rpm rotary shaking. Dissolved oxygen levels were measured with planar oxygen sensor spots and a Fibox 3 Oxygen Meter (PreSens, Regensburg, Germany) and maintained between 0 and 0.5 mg/L by daily replenishment with air injected through a 0.2 μm filter.

### RNA Extraction and Centrifugation

RNA was extracted from duplicate labeled and unlabeled microcosm sediments after 7 days of incubation using the RNA PowerSoil^®^ Total RNA Isolation Kit (Qiagen, Hilden, Germany). Isopycnic density gradient centrifugation was carried out according to Lueders (2015). Gradients were prepared with 5 ml of 2 g/ml cesium trifluroacetate (CsTFA; GE Healthcare, Munich, Germany), 185 μl formamide, and 1 ml of gradient buffer (0.1 M Tris-HCl, 0.1 M KCl, 1 mM EDTA) containing 1 μg of RNA. Gradients were loaded into 5.1 ml Quick Seal centrifuge tubes (Beckman Coulter, Indianapolis, United States), sealed, and spun on a VT1 65.2 rotor in an Optima XE90 ultracentrifuge (Beckman Coulter, Indianapolis, United States) at 125,000 g for ∼60 h. Seven fractions from each tube were collected according to Lueders (2015). Fraction density was measured by weighing known volumes. RNA was recovered by precipitation with 1 vol. of isopropanol, washed with cold 70% EtOH, and resuspended in 25 μl EB (Qiagen, Hilden, Germany). RNA from duplicate microcosms was ultra-centrifuged separately and selected fractions were pooled following RNA quantification.

### RNA Quantification

RNA recovered from fractions was quantified with RT-qPCR using the AccessQuick RT-PCR kit (Promega, Madison, WI, United States) and Ba519f and Ba907r universal 16S primers on an Mx3000p machine (Agilent, Santa Clara, CA, United States) as described by Glaubitz et al. (2009). rRNA standards were generated using the Riboprobe *in vitro* transcription kit (Promega, Madison, WI, United States) from plasmids containing a cloned 16S rRNA gene from *Methylobacterium* sp., and quantified with the Quant-iT RiboGreen RNA Assay Kit (Thermo Fisher Scientific, Waltham, MA, United States) on an Mx3000p (Agilent, Santa Clara, CA, United States). Each 40 μl RT-qPCR reaction contained: 20 μl 2x AccessQuick Master Mix, 0.4 μl 20 mg/ml bovine serum albumin, 0.2 μl 1/500 diluted SYBRgreen (Thermo Fisher Scientific, Waltham, MA, United States), 0.6 μl 1/500 diluted ROX, 10 μM each primer, and 2 μl of standard or unknown RNA. The temperature program was: 45°C reverse transcription for 30 min, initial denaturation of 95°C for 3 min, then 35 amplification cycles (30 s at 95°C, 30 s at 52°C, 30 s at 68°C), with a final extension step at 68°C for 5 min. After the run, a dissociation curve from 55 to 95°C was recorded to discriminate products from unspecific amplification products.

**FIGURE 1:**
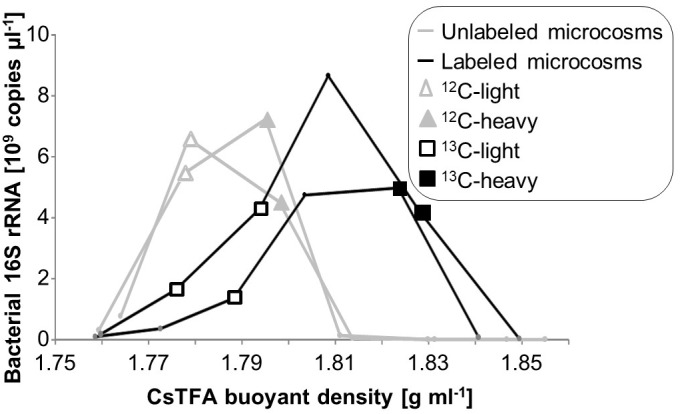
Quantitative distribution of rRNA in SIP gradients, as measured by RT-qPCR of bacterial SSU rRNA across buoyant density fractions. RNA fractions pooled from duplicate gradients for direct sequencing are given with respective symbols.

Based on quantitative rRNA profiles across gradients, selected RNA fractions from duplicate microcosms and gradients were pooled to obtain sufficient RNA amounts for linear amplification and sequencing (Figure 1). These pools were designated unlabeled light (^12^C-light, density 1.778–1.779 g ml^-1^), unlabeled heavy (^12^C-heavy, 1.795–1.798 g ml^-1^), labeled light (^13^C-light, 1.776–1.794 g ml^-1^) and labeled heavy (^13^C-heavy, 1.824–1.828 g ml^-1^). RNA amounts present in the heavy fractions of the ^12^C gradients were too low for transcriptome sequencing. Instead, the “heaviest” fractions from which sufficient RNA could be obtained from the ^12^C gradients were chosen, in order to control for possible shifts caused by physical characteristics of RNA such as GC content (Figure 1). Pooled RNA was concentrated via precipitation with isopropanol, washed with 70% EtOH, and resuspended in 15 μl EB (Qiagen, Hilden, Germany).

### Post-gradient RNA Treatment and Sequencing

Linear amplification was performed on a subset of each of the four RNA pools using the MessageAmp II Bacteria Kit (Thermo Fisher Scientific, Waltham, MA, United States) according to manufacturer instructions. RNA was quantified and purity-checked using a NanoDrop ND-1000 spectral photometer (VWR, Ismaning, Germany) and a 2100 Bioanalyzer (Agilent, Santa Clara, CA, United States) with the RNA 6000 Nano LabChip. Library preparation with input templates ≥20 ng of total RNA was performed with the TruSeq^®^ Stranded mRNA HT technology High Sample kit according to manufacturer protocols. The generated libraries were quality checked with the 2100 Bioanalyzer (Agilent) using a DNA 1000 LabChip, and quantified with the Qubit^®^dsDNA HS Assay Kit on a Qubit machine (Thermo Fisher Scientific, Waltham, MA, United States). A 1% PhiX v3 control library spike-in (Illumina, San Diego, CA, United States) was added to the library before cluster generation. Cluster generation and sequencing were performed on the NextSeq500 sequencing system (Illumina, San Diego, CA, United States) using a high-output paired-end 150 cycle (2x 150 PE) by IMGM Laboratories GmbH (Munich, Germany). All sequencing raw data generated in this study as well as sequences representing important 16S OTUs and functional transcripts have been submitted to the NCBI sequence read archive (SRA) via the Gene Expression Omnibus under the accession number GSE119644.

### Data Handling

An in-house bioinformatics pipeline was developed for transcriptome-SIP data handling. Removal of sequencing adapters and merging of paired-end sequences was performed with Adapterremoval (Lindgreen, 2012) with default settings except: trim ambiguous bases (N) at 5′/3′ termini, trim bases at 5′/3′ termini with quality scores ≤15 and minimum read length of 50. Deconseq (Schmieder and Edwards, 2011) was used to remove PhiX sequences. RNA was sorted into 16S, 23S, and non-rRNA using SortmeRNA v2 (Kopylova et al., 2012). The Biopieces framework (Hansen et al., 2008) was used for command line processes. Non-rRNA tags were compared against the NCBI-nr (downloaded February 2016), COG (Galperin et al., 2014), and KEGG v58.1 (Kanehisa et al., 2015) databases using DIAMOND (Buchfink et al., 2015). Non-rRNA analysis was performed using MEGAN 5 (Huson et al., 2007), which uses a lowest common ancestor (LCA) algorithm to assign reads to phylogenetic or functional groups, which places transcripts at the most specific level possible (Huson et al., 2007).

16S OTU assignment was performed using QIIME v1.9.1 (Caporaso et al., 2010). Preprocessing of reads identified as 16S by SortmeRNA was done with the split_libraries_fastq.py (quality = 2, *p* = 0.01), truncate_reverse_primer.py, identify_chimeric_seqs.py (method: usearch61), and filter_fasta.py scripts. Minimum Phred score was 2 (Bokulich et al., 2013). OTU calling was performed with a subsampled open-reference based method using RDP classifier (Wang et al., 2007) using the Silva database release 123 (Quast et al., 2013). For this, the scripts pick_open_reference_otus.py (97% similarity) and filter_otus_from_otu_table.py were used. 16S analysis and ordination plotting were performed in R (R Core Team, 2013) using the phyloseq package (McMurdie and Holmes, 2013). Significance tests were performed in Microsoft Excel 2010 using the Student’s *t*-test (paired, two-tailed).

### Calculation of Transcript Enrichment

Enrichment factors (EFs) were calculated as previously done in rRNA-SIP (Kramer et al., 2016; Zhang and Lueders, 2017) using the following equation:

$$\text{EF}=\left({\text{heavy}}^{13}\text{C}/{\text{light}}^{13}\text{C}\right)\text{-}\left({\text{heavy}}^{12}\text{C}/{\text{light}}^{12}\text{C}\right),$$

where “heavy ^13^C” and “light ^13^C” were the relative abundance of a specific rRNA taxon or mRNA transcript – assigned to the same KEGG/COG category – in heavy and light fractions of the ^13^C-toluene gradients, respectively, and “heavy ^12^C” and “light ^12^C” the same for respective ^12^C-control gradients. Positive values indicate isotopic labeling of a transcript; negative enrichment indicates more frequent detection in the light fraction of ^13^C-RNA.

### Linear Amplification Testing

A direct test of the effect of linear amplification on the proportion of mRNA in the total RNA was conducted in a SIP-independent manner using total RNA from *P. aeruginosa* and RT-qPCR of housekeeping genes. Further details can be found in the Supplementary Material.

## Results

### Total RNA Centrifugation

Total RNA extracted after 7 days of microcosm incubation and active degradation of toluene was subjected to isopycnic centrifugation. A clear shift in rRNA distribution over density gradients was observed between microcosms provided with ^13^C-labeled vs. unlabeled toluene (Figure 1). As previously also observed for DNA (Táncsics et al., 2018), the small amounts of RNA remaining in light fractions of ^13^C gradients suggested a community highly enriched in active toluene-degraders. Likewise, very little rRNA was present from unlabeled microcosms at a density >1.81 g ml^-1^. This complicated the selection of appropriate control fractions for sequencing, as our study, unlike most previous RNA-SIP studies, could not rely on post-gradient PCR amplification. Thus, we selected total RNA from the “heavier” and “lighter” slopes of unlabeled RNA (<1.78; >1.79 g ml^-1^, respectively) for sequencing of control fractions (Figure 1), in order to identify possible intrinsic density shifts of transcript pools caused by physical properties of RNA such as GC content.

### Composition of Total RNA-Seq Libraries

**FIGURE 2:**
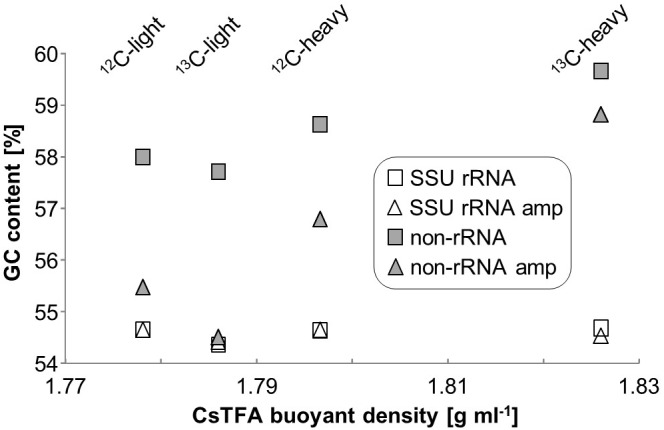
GC content of unamplified and amplified RNA-seq libraries of RNA-SIP buoyant density fractions.

A total of 279 million paired-end reads were obtained by RNA-seq across the eight libraries (4 unamplified, 4 which underwent linear amplification) with an average of ∼30 million per library. After QC, a total of about 1 million reads could be identified as mRNA using the KEGG or COG databases (Table 1). Total SSU and LSU (large subunit) rRNA reads accounted for 35 ± 3 and 64 ± 3% of sequencing reads, respectively. The ratio of non-rRNA reads was slightly but significantly increased in linearly amplified sequencing libraries (*p* = 0.023). GC-content of SSU rRNA reads appeared unchanged across gradient densities, and was also not significantly affected by linear amplification (Figure 2). However, a slight increase in average GC-content of non-rRNA reads (including mRNA) was observed with buoyant density (∼58% GC at ∼1.78 g ml^-1^; 59.5% GC at ∼1.83 g ml^-1^, respectively). Moreover, linear amplification significantly reduced the GC-content of non-rRNA reads by an average of 2.1 ± 0.9% compared to unamplified libraries (*p* = 0.025).

**Table 1:** Number of RNA sequencing reads and proportion of reads identified as rRNA and non-rRNA in RNA-seq libraries of density-resolved total RNA from SIP gradients.

	Starting reads (10^6^)	SSU rRNA reads (%)	LSU rRNA reads (%)	non-rRNA (%)	KEGG mRNA tags (10^3^)	COG mRNA tags (10^3^)
^12^C Light	42.5	31	68	0.6	101	110
^12^C Heavy	30.3	37	62	0.8	93	101
^13^C Light	37.2	37	60	1.8	150	171
^13^C Heavy	46.5	33	66	0.6	102	136
^12^C Light Amp	29.9	40	58	1.4	151	162
^12^C Heavy Amp	53.6	31	67	1.0	192	211
^13^C Light Amp	15.7	34	62	2.2	88	101
^13^C Heavy Amp	23.4	34	65	1.2	122	131

### Reads Enriched in ^13^C-Labeled rRNA

**FIGURE 3:**
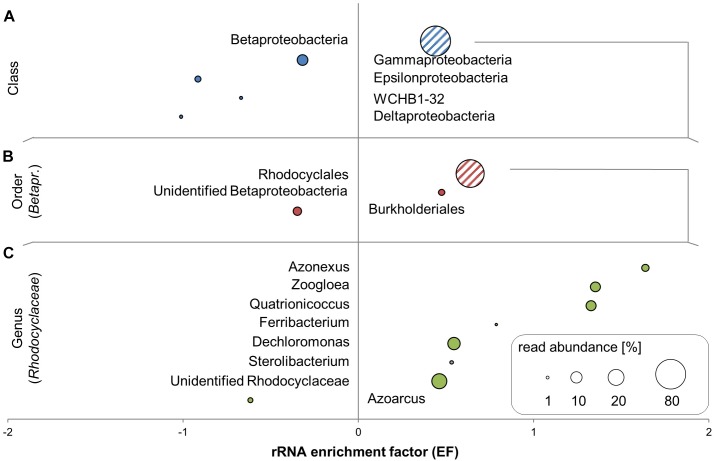
^13^C-enriched bacterial taxa identified by SIP in unamplified RNA of toluene-degrading microcosms. Rankings of SSU rRNA enrichment factors (EFs) are resolved at the class-level **(A)**, for orders within *Betaproteobacteria* **(B)**, and for genera within *Rhodocyclaceae* **(C)**. EFs are shown in combination with relative read abundances averaged across all RNA-seq libraries. Groups shown are those with >1% average read abundance at each level.

Nearly all SSU rRNA sequences in the libraries were bacterial, with a maximum of 0.09% of reads in libraries matching to eukaryotic rRNA and no reads matching archaeal rRNA. The community was overwhelmingly comprised of *Proteobacteria* (Supplementary Table S1), with an average of 98% of SSU reads, with only small contributions of *Bacteroidetes* (avg. 1.8%) and other phyla (avg. 0.6%). Most of the transcripts within the *Proteobacteria* were affiliated to the *Betaproteobacteria*; of these the majority belonged to members of the *Rhodocyclaceae* (Figure 3). Five of the six most abundant genera in total rRNA (unidentified *Rhodocyclaceae, Dechloromonas, Pseudomonas, Quatrionicoccus, Zoogloea*, and *Azonexus*) belonged to the *Rhodocyclaceae*. All genera within the *Rhodocyclaceae* were strongly enriched in ^13^C-labeled RNA, with the exception of reads affiliated to *Azoarcus* spp. (Figure 3); no labeling appeared in members outside of this family. About 21% of the total rRNA reads were within the *Rhodocyclaceae*, whose more precise affiliation remained unclear. rRNA of *Pseudomonas* spp. comprised an average 10% of the communities and appeared unlabeled.

Considerable differences in taxon-level abundances were found between unamplified and amplified libraries (Supplementary Table S1). For example, *Dechloromonas* spp. averaged at 11.4% of the unamplified community but at 18.3% after amplification, while *Zoogloea* rRNA was detected at 13.4% in the unamplified community and at 6% in the amplified.

### Functional Transcripts Enriched in ^13^C-Labeled RNA

**FIGURE 4:**
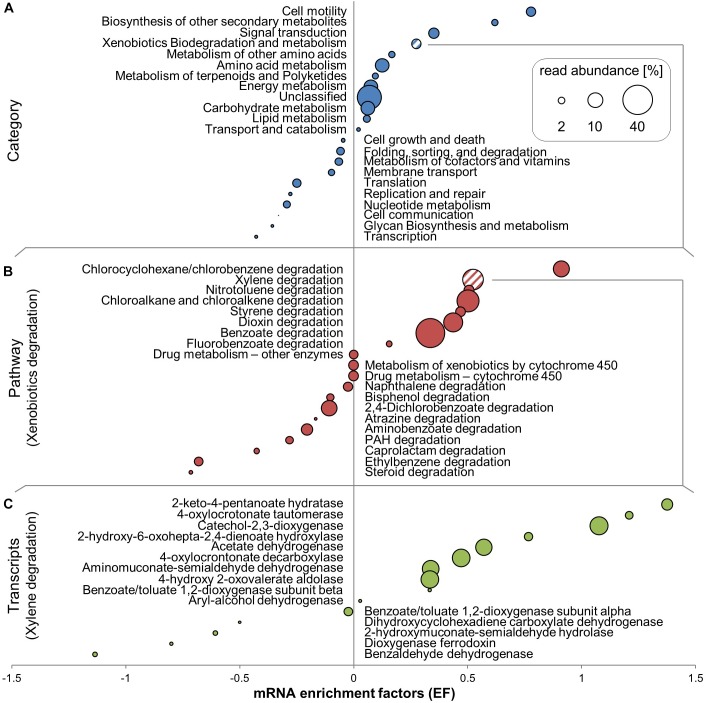
^13^C-enriched mRNA transcripts identified by SIP in unamplified RNA of toluene-degrading microcosms. Rankings of mRNA enrichment factors (EFs) are resolved at the level of KEGG categories **(A)**, pathways within the xenobiotics biodegradation and metabolism category **(B)**, and individual transcripts within the xylene degradation pathway **(C)**. EFs are shown in combination with relative read abundances averaged across all RNA-seq libraries. Individual transcripts shown are those with ≥ 20 total reads.

The number of non-rRNA reads identified as mRNA was between 88 and 192 × 10^3^ reads per library (Table 1). The transcript ratios discussed in this section are given as a percentage of those reads, not of total non-rRNA, which also included tRNA and other RNA types. The most abundant category of functional transcripts could not be further identified using KEGG and COG databases (avg. 30% of reads using KEGG and 26% of reads using COG). Among those reads which could be further identified, transcripts linked to genes involved in carbohydrate, amino acid, and energy metabolism were most abundant and were all enriched in ^13^C-RNA, as calculated via EFs for the different gene categories, pathways and transcripts (Figure 4).

Cell motility was the transcript category most highly enriched in ^13^C-RNA (Figure 4A). Many transcripts linked to motility-associated genes were highly abundant in mRNA (e.g., Flagellin at ∼2.65% of all transcripts in KEGG annotation), and almost all were enriched in ^13^C-labeled RNA. Supplementary Table S2 shows the phylogenetic affiliation of transcripts of the *fliC* gene based on MEGAN 5’s LCA algorithm. Between 35 and 40% of these transcripts could only be assigned at the phylum level, at which nearly all (96–100%) belonged to the *Proteobacteria*. Some could be specifically assigned at the species level, and 10–20% of all transcripts linked to *fliC* were assigned to *Azovibrio restrictus*.

The second most clearly labeled transcript category was linked to genes coding for biosynthesis of secondary metabolites (Figure 4A). The main ^13^C-enriched transcripts in this group were identified as genes coding for a catalase-peroxidase (K03782), which averaged at over 1000 copies per library. Labeling observed for the most abundant COG categories was generally consistent with KEGG results (Supplementary Figure S1), but biodegradation pathways are not resolved as a separate category in this database. The COG category “Secondary metabolite biosynthesis” includes similar pathways as the respective KEGG category, but also includes transcripts linked to genes involved in BTEX degradation, such as phenol hydroxylase and phenol-2-monooxygenase, which were highly enriched in ^13^C-RNA. Signal transduction was the third most labeled transcript category (Figure 4A). Approximately 20% of transcripts assigned there were for the *fliC* gene, which besides its role in motility is also part of the two-component signaling system in KEGG.

#### Xenobiotic Degradation Transcripts

“Xenobiotics degradation” was the fourth most clearly labeled transcript category in KEGG (Figure 4A), though about half of the pathways in that category were themselves not enriched in ^13^C-RNA (Figure 4B). It should be noted that many of the pathways expressed and enriched in ^13^C shared enzymes – for example, phenol hydroxylase (K16249), which can be involved in chlorocyclohexane and chlorobenzene degradation, benzoate degradation, toluene degradation, and in the degradation of aromatic compounds in general. Transcripts of the benzoate degradation pathway were the most abundant, though not the most highly labeled (Figure 4B).

Transcripts of catechol-2,3-dioxygenase (C23O), an enzyme previously associated with central pollutant metabolism at the investigated site (Táncsics et al., 2010, 2012, 2013), were identified as very abundant and enriched in ^13^C in KEGG (Figure 4C). Taxonomic association of these C23O transcripts was done using MEGAN 5 (Supplementary Table S3). Of the transcripts that could be assigned at the family level, the highest percent belonged to the *Rhodocyclaceae* (avg. 7.8%), followed closely by *Pseudomonadaceae* (avg. 6.5%). However, most of these transcripts could not be assigned more specifically than at the phylum level. Transcripts of the catechol-1,2-dioxygenase gene (K03381), on the other hand, were barely present, appearing only in very low read numbers (∼1) per library.

Amongst initial hydrocarbon degradation mechanisms, transcripts of the phenol hydroxylase gene (*dmpL*; K16243) were highly abundant and labeled, with transcript numbers of up to 160 per library. In contrast, transcripts of genes coding for toluene monooxygenase subunits (K15760-K15764, which may code for toluene-2,3, or 4-monooxygenases) were present in low read numbers (∼15), with varying amounts of labeling. Transcripts linked to genes coding for benzylsuccinate synthase (*bssA*; K07540) were present in all treatments but not very abundant (∼20 reads) and not labeled.

#### Transcripts of Respiratory Pathways

Transcripts of genes required for prokaryotic oxidative phosphorylation were present in the microoxic microcosms, but the majority were unlabeled. The full operon of *nuoA-N*, coding for Type I NADH dehydrogenase, was expressed, while no transcripts of the *ndh* operon for Type II NADH dehydrogenase were detected. Complete pathways of both denitrification and dissimilatory nitrate reduction to ammonium (DNRA) were transcribed, but the only transcript enriched in ^13^C-RNA found in these pathways codes for *NapA* (K02567). We were also interested in whether the putative NO-dismutase genes previously detected at the site (Zhu et al., 2017) would be expressed during microaerobic toluene degradation. For this, all mRNA transcripts assigned to “*Candidatus* Methylomirabilis oxyfera” (between 0 and 62 reads per library) were compared to the NCBI-nr database via nucleotide BLAST. All transcripts annotated as canonical nitric oxide reductases belonging to “*Ca.* Methylomirabilis oxyfera” actually matched more closely to the putative nitric oxide dismutase (*nod*). Up to 21% of all transcripts annotated as nitric oxide reductase subunit B (*norB*; K04561) were putative *nod* transcripts. These *nod* transcripts appeared in read numbers of ten or fewer, so EF calculation was not robust.

### Effect of Linear Amplification

**FIGURE 5:**
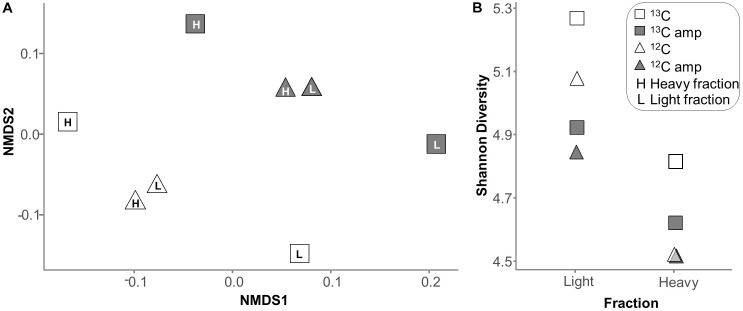
**(A)** NMDS ordination of profiles of functional transcript reads as identified in unamplified and amplified RNA-seq libraries of RNA-SIP fractions using KEGG. **(B)** Shannon diversity of SSU rRNA reads in the same libraries.

Linear amplification increased RNA amounts by at least 300-fold. NMDS plots of functional transcript pools (Figure 5A and Supplementary Figure S2B) and of SSU rRNA profiles (Supplementary Figure S2A) in the 8 treatments were used to delineate the impact of linear amplification on total RNA-seq libraries, and on overall labeling patterns. Within the unamplified or amplified data sets, the separation between profiles was consistent with expectations for a typical SIP experiment. For example, heavy and light profiles for the control (^12^C) microcosm were much more similar, while those of the labeled (^13^C) microcosm were clearly separated, mainly on NMDS axis 1 (Figure 5A). This separation was observed for both unamplified and amplified libraries. However, linear amplification itself seemed to introduce a further discriminant vector to overall transcript pools, showing that this treatment changed the community profile. A similar skewing pattern was also observed for SSU rRNA (Supplementary Figure S2A).

Not unexpectedly, overall transcript diversity was always lower in heavy than in light fractions (Figure 5B). Diversity of transcript libraries after linear amplification was consistently though not significantly (*p* = 0.073) decreased, suggesting that the amplification process could leave behind some specific transcripts or over-amplify others. Rare transcripts with copy numbers less than ten appeared with equal frequency in amplified and unamplified libraries (Supplementary Figure S3). Additionally, predicted Chao1 richness perfectly matched the richness observed (not shown), suggesting an adequate sequencing depth for sufficient community coverage.

In addition, we conducted a SIP- and sequencing-independent lab experiment with pure culture RNA of *P. aeruginosa*, to directly test whether linear amplification increased the proportion of mRNA in the total RNA. RT-qPCR results of housekeeping gene transcripts did not indicate that linear amplification systematically increased mRNA ratios (Supplementary Figure S4). In fact, two of the three housekeeping genes tested had lower transcript/total RNA ratios after linear amplification than before, while one remained unchanged.

## Discussion

### Methodological Considerations

This study demonstrates the value of total-RNA SIP (also termed transcriptome-SIP) in providing targeted insights into microbial activities in processes such as pollutant degradation, on both taxonomic and functional levels. However, the level of detail clearly depended on the number of mRNA reads obtained from gradient fractions. The amount of non-rRNA reads was between 0.6 and 2.2% in our libraries, but this small proportion was not unexpected for total RNA-seq and is consistent with the percent non-RNA or putative mRNA found in other total-RNA-seq studies (Supplementary Table S4). Most transcriptomic studies use mRNA enrichment (eukaryotes) or rRNA depletion (prokaryotes) to increase the depth of mRNA sequencing. However, our interest in SSU-based microbial community composition and desire to avoid potential skewing effects motivated a depletion-free, total-RNA approach. This made deep sequencing necessary for adequate non-rRNA coverage, preventing the sequencing of true biological replicates for RNA fractions. However, the inclusion of both amplified and unamplified libraries, in which overall labeling patterns were generally consistent (Figure 5 and Supplementary Figure S2 and Supplementary Tables S1–S3), provided a certain level of confidence to this end. A further cautioning statement needs to be added with reference to the comparably late time point in microcosm incubation (7 days) chosen for transcriptome-SIP analysis. This was motivated by our need for abundant and highly ^13^C-labeled RNA in this primary proof-of-concept study, while we are aware that certain patterns of transcript labeling during initial phases of toluene catabolism may have been missed, due to the possibly more rapid turnover of distinct mRNA species.

The interpretation of SIP data strongly relies on comparing sequencing results between ^12^C and ^13^C-treatments, and across similar ranges of buoyant densities (Whiteley et al., 2007). This was not fully possible in the present study, mainly because we could not rely on post-gradient PCR amplification of ^12^C-RNA recovered from heavy fractions and vice versa. Direct sequencing of total RNA and parallel linear amplification necessitated a certain minimum amount (∼120 ng) of total RNA recovered after fractionation. To increase yields for low-RNA fractions, several fractions of similar density across duplicate centrifugation gradients were pooled (Figure 1). In essence, RNA from all meaningful fractions of the density-resolved total RNA obtained were sequenced from both ^12^C and ^13^C gradients, which was the best that could be accomplished considering methodological limitations. While the lack of sequencing replicates precludes statistical calculations or differential expression analysis that have been previously used in amplicon-based SIP studies (Pepe-Ranney et al., 2016), the EFs based on differences in labeled and control microcosms offer a semi-quantitative way to interpret labeling (Kramer et al., 2016; Zhang and Lueders, 2017). Nevertheless, the total amounts of RNA recovered from gradient fractions and the net costs of transcriptome sequencing will remain a major limitation of transcriptome-SIP work in the future.

### Effect of Linear Amplification

Linear amplification is one potential strategy to alleviate the technical limitations involved in total RNA-seq following SIP gradients. We compared this to direct sequencing in the present study. Linear amplification increased RNA amounts, but also decreased GC content in non-rRNA (Figure 2), decreased library diversity (Figure 5B), and caused profound changes to community profiles for SSU rRNA and functional transcripts (Figure 5A and Supplementary Figure S2). Linearly amplified libraries contained a much higher percentage of small (<50 bp) read fragments which were removed early in the bioinformatic pipeline. Previous studies have also found changes in transcript ratios when using various linear amplification protocols (Nygaard et al., 2003; Degrelle et al., 2008), which may be due to inefficiency or bias in the initial reverse transcription step (Levesque-Sergerie et al., 2007). The equal numbers of rare transcripts in amplified and unamplified libraries (Supplementary Figure S3) indicate that the amplification process did not simply miss less common transcripts, but must have selected based on another characteristic, perhaps GC-content (Figure 2).

While the proportion of non-rRNA was somewhat increased by linear amplification (Table 1), this increase was too small to provide tangible benefits in the face of the negative effects caused by the treatment. Our independent test based on RT-qPCR targeting *P. aeruginosa* housekeeping genes did not indicate an increase in mRNA ratios as a result of linear amplification (Supplementary Figure S4). Thus we cannot confirm the idea that linear amplification could increase the proportion of mRNA to total RNA reads in sequencing libraries in a meaningful manner (Cao et al., 2010). Because of the marked skewing effects observed in overall library composition (Figure 5A and Supplementary Figure S2), we recommend that linear amplification should be used only if total RNA yield is too low for direct analysis, and samples studied using linear amplification should only be compared to samples treated likewise.

### Taxonomic Insights via rRNA of Labeled Degraders

Since contaminated aquifers are well-known to harbor not only bacteria, but also archaea and microeukaryotes (Griebler and Lueders, 2009), the lack of respective rRNA in our libraries came as a surprise. Possibly, the limited availability of oxygen in our microoxic microcosms as well as the substantial toluene loads (∼1 mM; Táncsics et al. (2018)) contributed to selection of specific bacterial degraders during SIP incubation. *Betaproteobacteria* have been reported previously to be prevalent in oxygen-limited, aromatic hydrocarbon-contaminated groundwater environments (Fahy et al., 2006). *Rhodocyclaceae* in particular are known to be important at this site, both from previous samplings (Táncsics et al., 2012, 2013) and from DNA-SIP of the same microcosms as used in this study (Táncsics et al., 2018). Members of the *Rhodocyclaceae* can degrade aromatics both aerobically and anaerobically (Song et al., 2001; Chakraborty et al., 2005). *Dechloromonas aromatica*, for example, can degrade several aromatic compounds using oxygen, nitrate, perchlorate, or chlorate as electron acceptors (Chakraborty et al., 2005). *Dechloromonas* rRNA was very abundant and clearly enriched in ^13^C-rRNA, despite being represented by less than 1% of the DNA amplicons sequenced in DNA-SIP of the same microcosms (Táncsics et al., 2018). Since labeling of DNA requires cellular replication, while RNA labeling can occur independent of cellular growth (Manefield et al., 2002), this could indicate that *Dechloromonas* was metabolically active despite slow growth during microoxic SIP incubation. Alternately, this differential representation could be a result of primer bias during the production of PCR amplicons for sequencing. Primer bias was even more clearly apparent for the genus *Azonexus*, which was abundant (∼6%; Supplementary Table S1) and highly enriched in ^13^C-rRNA, while not detected at all in DNA amplicons (Táncsics et al., 2018).

*Quatrionicoccus* spp. (a genus within the *Rhodocyclaceae* – originally called *Quadricoccus* but reclassified as *Quatrionicoccus*; Tindall and Euzéby, 2006) – were first isolated from activated sludge biomass in an enhanced biological phosphorus removal reactor with cycling aerobic/anaerobic conditions (Maszenan et al., 2002). Members of this genus are considered to be strict aerobes. This genus comprised an average 10% of the rRNA reads and was highly labeled, indicating effective toluene metabolism under microoxic conditions. This was consistent with labeling results from DNA-SIP of the same microcosms (Táncsics et al., 2018). The genus is also highly abundant in Siklós groundwater itself (Farkas et al., 2016). Before this work, members of the genus have not been described as hydrocarbon degraders. This contrasts other members of the *Rhodocyclacaeae* such as *Zoogloea* spp. which were clearly identified as labeled in both rRNA and mRNA in the present study, and which have been frequently reported as aerobic degraders of aromatic hydrocarbons (Jechalke et al., 2013; Farkas et al., 2015).

In contrast, other microbes of the BTEX contaminated sediment were abundant in RNA but without any indication of labeling, including members of the *Pseudomonadaceae* and *Comamonadaceae*, which are well-known to degrade hydrocarbons (Fahy et al., 2006; Wald et al., 2015), under either aerobic (Martinez-Lavanchy et al., 2010) or nitrate-reducing conditions (Sun and Cupples, 2012). Thus in our experiment, they must have been active and producing transcripts, potentially thriving on electron donors introduced with the original sediment inoculum, but not receiving labeled carbon from the added toluene.

### Functional Insights via Labeled mRNA

The combination of stable isotope probing (SIP) with metatranscriptomic sequencing provided information on a whole range of functional transcripts produced by active degraders during incubation. Here, we discuss transcript labeling patterns which appeared most relevant to us in understanding degrader activity under microoxic conditions. Transcripts linked to genes coding for proteins involved in cell motility were the most highly labeled group in both KEGG and COG analyses (Figure 4A and Supplementary Figure S1). The importance of chemotaxis of degraders toward aromatics, including BTEX, is well-known (Krell et al., 2013). In highly contaminated systems, motile cells have advantages not only in moving toward contaminant sources, but also in escaping toxically high local concentrations (Parales et al., 2000). Still, the apparent importance of motility amongst degraders in our agitated SIP microcosms came as a surprise, and could potentially be connected to a lower availability of toluene or oxygen during the late phase of degradation (Táncsics et al., 2018), triggering a motility response (Lacal, 2017).

Transcripts of genes coding for flagellin, the principle component of bacterial flagella, were the most abundant of the transcripts linked to motility and were highly ^13^C-enriched. Between 10 and 20% of all transcripts of the *fliC g*ene were assigned to *Azovibrio restrictus* (Supplementary Table S2), a N_2_-fixing microaerophile within the *Rhodocyclaceae* (Reinhold-Hurek and Hurek, 2000), while only ∼0.1% of 16S rRNA reads were affiliated to *Azovibrio* sp. (Supplementary Table S1). We find it highly unlikely that an organism so rare within the community could have contributed so much of one of the most abundant and highly labeled transcripts in our microcosms. Either lateral gene transfer, or the lack of more appropriate reference genome data for other members of the *Rhodocyclaceae*, such as *Azonexus* spp., could potentially explain this finding. No other single species contributed more than 1.6% of transcripts of *fliC* within each sample, even known motile toluene degraders such *Dechloromonas* (Chakraborty et al., 2005) or *Zoogloea spp.* (Farkas et al., 2015), which were much more abundant in rRNA reads than *Azovibrio*.

#### Xenobiotic Degradation Transcripts

The relatively high copy numbers and labeling of transcripts for the phenol hydroxylase gene and the low numbers or complete absence of other possible oxygen-dependent toluene-activating gene transcripts indicated that phenol hydroxylase was the primary driver of toluene activation in these microcosms. Phenol hydroxylase has a broad substrate specificity for monoaromatics and its gene has been previously found in microoxic BTEX-contaminated groundwater (Da Silva and Corseuil, 2012) and used as a biomarker for aerobic aromatic degradation (Nebe et al., 2009). However, it is also possible that transcripts of other genes in upper pathways of toluene catabolism were transcribed at earlier time points of microcosm incubation, but may have been subject to more rapid turnover and decay.

Catechol-2,3-dioxygenase (C23O) is an extradiol dioxygenase which has long been used as a conserved functional marker gene for aerobic aromatic degraders (Táncsics et al., 2010, 2012, 2013). Kukor and Olsen (1996) found that C23O in the subfamily 1.2.C of extradiol dioxygenases efficiently used low amounts of dissolved oxygen, and likely evolved in environments with low oxygen and substrate concentrations. Considering its frequent presence in contaminated environments (Hendrickx et al., 2006; Táncsics et al., 2012) and the previously seen high diversity at this site (Táncsics et al., 2010, 2012, 2013), it is not surprising that C23O was highly expressed and the related transcripts highly ^13^C-labeled in SIP microcosms. The majority of these transcripts could only be identified to the phylum level (Supplementary Table S3). This may be because gene sequences are so similar across different lineages that affiliation by MEGAN’s lowest-common-ancestor algorithm stopped at a higher level. A more likely explanation is the lack of more specifically assigned reference sequences in the KEGG database, since most C23Os could not be linked to any cultured bacterium.

Within the transcripts that could be assigned more specifically, *Rhodocyclaceae* were most prominent. This included matches to *Azovibrio restrictus*, previously found in hydrocarbon-contaminated soil (Yang et al., 2014) and to *Zoogloea oleivorans*, a facultative aerobic hydrocarbon-degrader (Farkas et al., 2015). Transcripts of the C23O gene affiliated to the *Pseudomonadaceae* were nearly as frequent as that of the *Rhodocyclaceae* (avg. 6.5%), although *Pseudomonadaceae* were clearly not isotopically enriched (Supplementary Table S3). Members of this family are well-known as aerobic degraders of BTEX compounds (Martinez-Lavanchy et al., 2010) and have long been known to harbor C23O (Kojima et al., 1961). The results of our labeling experiment suggest that they were not competitive in toluene degradation in the microcosms, or assimilated C from alternative substrates for biomass buildup.

Microoxic sedimentary systems may well contain anoxic micro-niches where anaerobic metabolism may take place. Furthermore, the daily replenishment of oxygen during microcosm incubation (Táncsics et al., 2018) resulted in periods of anoxia. For these reasons, we also searched for transcripts of marker genes of anaerobic aromatic degradation, i.e., benzylsuccinate synthase (Bss) (Von Netzer et al., 2016). While *bssA* was indeed transcribed in all microcosms, transcripts were not found labeled, indicating that anaerobic degraders were not competitive in the microcosms.

#### Transcripts of Respiratory Pathways

Genes involved in aerobic respiratory pathways were transcribed in low to moderate copy numbers. However, transcripts of genes coding for a Type I NADH dehydrogenase were present alone, although the majority of *Betaproteobacteria*, which dominated the system, are known to harbor genes for Type II as well (Marreiros et al., 2016). This may indicate an adaptation to microoxic conditions, as some obligate microaerophiles harbor only Type I (Smith et al., 2000), and expression of Type II in *E. coli* occurs only under fully oxic conditions (Spiro et al., 1989).

Despite not having added nitrate to microcosms, transcripts of genes coding for all steps of canonical denitrification and DNRA were detected. All related transcripts were unlabeled except for *napA*, which codes for a nitrate reductase adapted to low-nitrate conditions (Potter et al., 1999) and has been suggested to be involved in aerobic denitrification (Ji et al., 2015), indictating that such physiologies could have been relevant during microcosm incubation. Moreover, a previous study found abundant putative nitric oxide dismutase (*nod*) genes at this site (Zhu et al., 2017). NO-dismutation is hypothesized to provide a novel mechanism in reductive nitrogen cycling (Ettwig et al., 2012). The enzyme is proposed to dismutate NO into N_2_ and O_2_, thus producing intracellular O_2_ for the oxygenase-dependent activation of hydrocarbons (Ettwig et al., 2010; Zedelius et al., 2011). A recent metatranscriptomics study has indeed found both anaerobic and aerobic catabolic pathways to be expressed in a denitrifying, benzene-degrading enrichment culture dominated by members of the *Peptococcaceae*, although transcripts related to NO-dismutation were not detected (Atashgahi et al., 2018). Since the discovery of respective *nod* genes is still recent, it is likely that such sequence reads can still be misannotated as canonical NO-reductases (Nor) in databases. In the present study, transcripts from “*Ca.* Methylomirabilis oxyfera” that were annotated by KEGG as *nor*s more closely matched putative *nod* sequences (Zhu et al., 2017) when compared to the NCBI-nr database.

In summary, this study builds on the advantage of metatranscriptome sequencing to provide functional details about microbial activities in a given system, which may be hard to delineate with more traditional, targeted techniques like RT-PCR. At the same time, the combination with isotopic labeling adds a new process-targeted handle to the metatranscriptomics pipeline, with a potential to provide functional context even for constituents of the large fraction of unclassified transcripts, which were detected also in our study. Here, we confirm the central role of *Rhodocyclaceae* and the respective C23O pathways in toluene degradation in microoxic microcosms (Táncsics et al., 2018). In addition, we reveal the important role of phenol hydroxylase as well as gene categories outside of strict catabolism, such as cell motility, and also of different respiratory pathways in active degrader populations. In the future, this novel transcriptome-SIP approach can be extended to elucidate microbial activities and interactions in processes and systems beyond contaminated aquifers.

## Author Contributions

LB, AT, and TL designed the experiments. LB and GV analyzed the data, with specific contributions from BZ and MS. LB and TL wrote the initial manuscript draft with additions and revisions contributed from all authors.

## Conflict of Interest Statement

The authors declare that the research was conducted in the absence of any commercial or financial relationships that could be construed as a potential conflict of interest.
