# Supplementary material for: Transcriptome-Stable Isotope Probing Provides Targeted Functional and Taxonomic Insights Into Microaerobic Pollutant-Degrading Aquifer Microbiota
Source: Front Microbiol. 2018 Nov 13;9:2696. doi: 10.3389/fmicb.2018.02696 (PMC6243674; doi:10.3389/fmicb.2018.02696)
Supplement: Supplementary file 1 [file Data_Sheet_1.PDF]

Transcriptome-stable isotope probing provides targeted functional and taxonomic insights into microaerobic pollutant-degrading aquifer microbiota

Lauren M. Bradford, Gisle Vestergaard, András Táncsics, Baoli Zhu, Michael Schloter, Tillmann Lueders*

Supplementary material

Supplementary methods and results 2

Sequencing-independent testing of linear amplification by RT-qPCR 2

COG annotation of functional transcripts 3

Supplementary References 4

Supplementary Tables 5

Table S1: 5

Table S2: 6

Table S3: 7

Table S4 8

Supplementary Figures 9

Figure S1: 9

Figure S2: 10

Figure S3: 11

Figure S4: 12

Supplementary methods and results

Sequencing-independent testing of linear amplification by RT-qPCR

Pseudomonas aeruginosa was grown in LB broth at 37°C with shaking for 70 hours, and RNA extracted using a bead-beating phenol-chloroform protocol modified from (Schmitt et al., 1990). Cells were pelleted at 1700 x g for 3 minutes and the cell mass added to sterile screw-cap tubes containing a 1:1 mix of 0.1 mm and 0.7 mm zirconia/silica beads. Each tube received 300 µL AE (50 mM sodium acetate, 10 mM EDTA, pH 5.3), 200 µL phosphate buffer (200 mM $NaPO_4$, pH 5.6), 50 µL 20 % sodium dodecyl sulfate, and 450 µL acidic phenol (Carl Roth). Tubes were incubated at 65 °C for 10 minutes, bead beat at 6.5 m s^{-1} for 30 s, then centrifuged at 20800 x g for 5 min at 4 °C. Aqueous supernatants were transferred to Phase Lock Gel tubes (5Prime) and extracted with one volume acidic phenol/chloroform/isoamyl (Carl Roth). Nucleic acids were precipitated with 2 volumes of 30% polyethylene glycol, 1.6 mM NaCl solution by centrifuging at 20800 x g for 30 min at 4 °C, then washed with 70 % ethanol and resuspended in RNAse-free water. DNA was removed from extracts by treatment with RQ1 DNAse (Promega). Housekeeping genes *rpoD, rpoS,* and *gyrPA* were amplified by RT-PCR with PCR primers from Savli et al. (2003) or Qin et al. (2003) using the AccessQuick RT-PCR kit (Promega). The temperature program was: 45 °C reverse transcription for 45 min, initial denaturation of 95 °C for 5 min, then 35 amplification cycles (15s at 95 °C, 10s at 60 °C, 15s at 72 °C), with a final extension step at 72 °C for 7 min. Products were cloned using the pGEM-Easy plasmid kit (Promega) and *E. coli* JM109 competent cells. Clones were used to generate RT-qPCR standards via in vitro transcription, as above. Fresh RNA was extracted, and either amplified with the MessageAmp II Bacteria kit (Ambion) or kept untreated. Total RNA in amplified or unamplified subsamples was quantified with the Quant-iT RiboGreen RNA Assay Kit (Thermofisher) as above. Copy numbers of housekeeping genes were quantified by RT-qPCR on the Mx3000p using the AccessQuick kit using SYBR green dye, as above, with a final primer concentration of 100pM. The temperature program was: 45 °C reverse transcription for 25 min, initial denaturation of

95 °C for 5 min, then 45 amplification cycles (15s at 95 °C, 10s at 60 °C, 15s at 72 °C), with a dissociation curve from 55 °C to 95 °C recorded after the run. Ratios of housekeeping gene copy number to total RNA were calculated for comparison.

COG annotation of functional transcripts

Detailed results of functional transcript annotation using the COG database are illustrated in Fig. S1 and generally confirm the results obtained via the KEGG database. The most abundant group was "Unknown function" (26 %), equivalent to the "Unknown" category that made up 30 % of KEGG annotations. While the "unclassified" KEGG category showed a slight positive enrichment, "Unknown Function" in COG was unlabeled, reflecting some difference between which transcripts could be annotated by the two databases. For the most part, similar categories between the two databases showed similar patterns of enrichment (Fig. 4, Fig. S1). Cell motility was the most highly enriched category, followed by secondary metabolite biosynthesis. While COG does not have a category devoted to xenobiotic degradation, many of the transcripts involved in these metabolisms, for example phenol-2-monooxygenase, belong to the secondary metabolite biosynthesis category. "Energy metabolism" (KEGG) and "Energy production and conversion" (COG) were similarly abundant and labeled, likewise "Amino acid metabolism" and "Amino acid transport and metabolism". Categories related to replication, cell growth/cycling, transcription, and translation were unlabeled in both databases.

Supplementary References

Fortunato, C. S., and Huber, J. A. (2016). Coupled RNA-SIP and metatranscriptomics of active chemolithoautotrophic communities at a deep-sea hydrothermal vent. *ISME J.* 10, 1925-1938. doi:10.1038/ismej.2015.258.

Qin, X., Emerson, J., Stapp, J., Stapp, L., Abe, P., and Burns, J. L. (2003). Use of real-time PCR with multiple targets to identify Pseudomonas aeruginosa and other nonfermenting gram-negative bacilli from patients with cystic fibrosis. *J. Clin. Microbiol.* 41, 4312–4317. doi:10.1128/JCM.41.9.4312-4317.2003.

Radax, R., Rattei, T., Lanzen, A., Bayer, C., Rapp, H. T., Urich, T., and Schleper, C. (2012). Metatranscriptomics of the marine sponge Geodia barretti: tackling phylogeny and function of its microbial community. *Environ. Microbiol.* 14, 1308–1324. doi:10.1111/j.1462-2920.2012.02714.x.

Savli, H., Karadenizli, A., Kolayli, F., Gundes, S., Ozbek, U., and Vahaboglu, H. (2003). Expression stability of six housekeeping genes: a proposal for resistance gene quantification studies of Pseudomonas aeruginosa by real-time quantitative RT-PCR. *J. Med. Microbiol.* 52, 403–408. doi:10.1099/jmm.0.05132-0.

Schmitt, M. E., Brown, T. A., and Trumpower, B. L. (1990). A rapid and simple method for preparation of RNA from Saccharomyces cerevisiae. *Nucleic Acids Res.* 18, 3091-3092.

Schwab, C., Tveit, A. T., Schleper, C., and Urich, T. (2014). Gene expression of lactobacilli in murine forestomach biofilms. *Microb. Biotechnol.* 7, 347–359. doi:10.1111/1751-7915.12126.

Supplementary Tables

Table S1

Phylogenetic affiliation and percent abundance of SSU rRNA reads from taxonomic units recovered in RNA-seq libraries of density-resolved total RNA from SIP gradients. Isotope enrichment for taxa was calculated via EFs.

	Unamplified				Amplified				Enrichment (unamplified)	Average Abundance
	Unlabelled		Labelled		Unlabelled		Labelled			
	Light	Heavy	Light	Heavy	Light	Heavy	Light	Heavy		
Proteobacteria	97.9	98.3	95.4	99.9	97.7	98.1	94.7	99.8	0.04	97.7
Betaproteobacteria	81.0	84.7	62.8	93.2	80.8	83.0	66.1	90.3	0.44	80.2
Rhodocyclaceae	67.9	71.8	48.0	81.4	69.9	70.9	56.4	81.1	0.64	68.4
NA	20.0	22.1	15.9	24.8	20.9	22.6	19.3	22.0	0.46	21.0
Dechloromonas	11.3	12.4	8.3	13.7	18.9	21.3	10.8	22.1	0.55	14.8
Quatrionicoccus	12.6	12.2	6.3	14.5	9.7	4.9	5.7	14.1	1.33	10.0
Zoogloea	13.8	14.5	7.5	17.9	5.6	7.3	4.3	6.9	1.35	9.7
Azonexus	3.5	3.8	1.7	4.7	7.5	8.6	4.3	10.6	1.64	5.6
Azoarcus	2.2	1.7	4.4	0.7	3.0	1.3	8.5	1.1	-0.62	2.9
Sterolibacterium	1.6	2.1	1.2	2.2	1.4	2.4	1.0	1.7	0.53	1.7
Ferribacterium	1.0	1.0	0.6	1.2	1.1	1.1	0.6	1.1	0.79	1.0
Azovibrio	0.0	0.2	0.0	0.1	0.1	0.1	0.0	0.1	0.17	0.1
Comamonadaceae	9.1	8.2	11.1	6.1	6.4	6.3	6.8	4.3	-0.35	7.3
NA	5.9	5.5	7.7	4.1	4.4	4.4	4.7	2.9	-0.40	4.9
Acidovorax	0.8	0.7	0.9	0.5	0.6	0.6	0.6	0.4	-0.28	0.6
Polaromonas	0.5	0.4	0.6	0.2	0.2	0.2	0.3	0.1	-0.33	0.3
Gammaproteobacteria	9.7	6.7	15.4	5.8	13.9	10.3	22.6	8.7	-0.32	11.7
Pseudomonadaceae	9.2	6.3	14.1	5.7	13.5	9.9	21.6	8.6	-0.28	11.1
Pseudomonas	8.3	5.8	13.1	5.3	12.9	9.8	20.9	8.2	-0.30	10.5
NA	0.8	0.4	0.9	0.4	0.5	0.0	0.6	0.4	-0.07	0.5
Epsilonproteobacteria	5.6	5.3	13.3	0.5	1.4	2.9	2.9	0.2	-0.92	4.0
Campylobacteraceae	4.7	4.6	11.5	0.4	1.3	2.6	2.5	0.2	-0.95	3.5
Arcobacter	3.8	4.0	9.8	0.4	0.9	2.1	1.9	0.1	-1.03	2.9
Sulfurospirillum	1.0	0.6	1.7	0.1	0.4	0.6	0.6	0.1	-0.61	0.6
Others	2.1	1.7	4.6	0.1	2.3	1.9	5.3	0.2	-0.74	2.3

NA – Not affiliated below family level

Table S2

Abundance and phylogenetic assignment of *fliC* transcripts (coding for flagellin) recovered in RNA-seq libraries of density-resolved total RNA from SIP gradients. Abundances are given relative to the total number of *fliC* transcripts in a given library. Only groups averaging >0.5 % of fliC transcripts in unamplified samples are shown. Isotope enrichment for taxa was calculated via EFs.

	Unamplified				Amplified					
	Unlabeled		Labeled		Unlabeled		Labeled			
	Light	Heavy	Light	Heavy	Light	Heavy	Light	Heavy	Enrichment (unamp)	Average Abundance
Bacteria	100.0	100.0	100.0	100.0	100.0	100.0	100.0	100.0	0	100
Proteobacteria	99.2	99.4	95.9	99.8	99.1	99.6	97.1	99.9	0.037164	98.6
Betaproteobacteria	59.3	53.4	36.2	60.1	50.8	62.2	37.1	66.2	0.759078	52.2
Burkholderiales	6.7	4.9	5.7	3.1	3.3	3.4	3.1	2.8	-0.18418	5.1
Comamonadaceae	5.6	4.0	3.9	2.3	2.9	2.9	2.3	2.3	-0.13155	4.0
Acidovorax	0.9	1.1	1.3	0.5	0.4	0.4	0.8	0.4	-0.84297	0.9
Acidovorax sp. KKS102	0.8	0.7	1.0	0.4	0.4	0.4	0.2	0.3	-0.43885	0.7
Rhodocyclales	39.4	34.2	20.0	40.3	33.1	43.0	22.1	47.8	1.14753	33.5
Rhodocyclaceae	39.4	34.2	20.0	40.3	33.1	43.0	22.1	47.8	1.14753	33.5
Azoarcus	0.9	1.2	0.3	0.7	1.6	2.0	1.3	1.2	0.967009	0.8
Azoarcus sp. KH32C	0.6	0.8	0.3	0.6	1.5	1.9	1.3	0.9	0.227442	0.6
Azovibrio	16.1	17.5	10.4	19.8	15.1	18.3	10.0	20.9	0.81703	16.0
Azovibrio restrictus	16.1	17.5	10.4	19.8	15.1	18.3	10.0	20.9	0.81703	16.0
Deltaproteobacteria	0.6	0.8	1.6	0.3	0.1	0.1	0.8	0.1	-1.1363	0.8
Desulfuromonadales	0.6	0.7	1.3	0.1	0.1	0.1	0.4	0.1	-1.0844	0.7
Geobacteraceae	0.6	0.7	1.3	0.1	0.1	0.1	0.4	0.1	-1.0844	0.7
Geobacter	0.6	0.7	1.3	0.1	0.1	0.1	0.4	0.1	-1.0844	0.7
Geobacter metallireducens	0.3	0.6	0.9	0.1	0.0	0.1	0.2	0.0	-2.17973	0.5
Epsilonproteobacteria	3.5	4.6	17.8	0.9	12.1	7.5	30.8	2.5	-1.27913	6.7
Campylobacterales	3.4	4.6	17.6	0.9	11.9	7.5	30.4	2.5	-1.30563	6.6
Campylobacteraceae	2.8	3.7	14.2	0.9	8.9	6.5	25.2	2.2	-1.23429	5.4
Arcobacter	2.4	2.6	10.3	0.5	6.4	4.6	20.2	1.5	-1.04544	3.9
Sulfurospirillum	0.4	0.9	3.3	0.3	2.2	1.6	5.0	0.7	-2.11999	1.2
Helicobacteraceae	0.5	0.7	2.9	0.0	2.5	0.7	1.0	0.2	-1.51715	1.0
Sulfuricurvum	0.3	0.3	1.7	0.0	1.7	0.2	4.0	0.1	-0.7965	0.6
Sulfuricurvum kujiense	0.3	0.3	1.6	0.0	1.6	0.2	2.9	0.1	-0.7965	0.6
Gammaproteobacteria	1.0	0.7	1.6	0.1	0.3	0.5	2.9	0.0	-0.63152	0.9
Pseudomonadales	0.6	0.5	1.0	0.1	0.3	0.4	0.4	0.0	-0.67377	0.5
Pseudomonadaceae	0.6	0.5	1.0	0.1	0.3	0.4	0.4	0.0	-0.67377	0.5
Pseudomonas	0.6	0.5	1.0	0.1	0.3	0.4	0.4	0.0	-0.67377	0.5
Spirochaetes	0.3	0.4	1.6	0.1	0.6	0.2	0.4	0.0	-1.02376	0.6
Spirochaetia	0.3	0.4	1.6	0.1	0.6	0.2	2.1	0.0	-1.02376	0.6
Spirochaetales	0.3	0.4	1.6	0.1	0.6	0.2	2.1	0.0	-1.02376	0.6
Spirochaetaceae	0.3	0.4	1.6	0.1	0.5	0.2	2.1	0.0	-1.02376	0.6

Table S3

Abundance and phylogenetic assignment of catechol-2,3-dioxygenase transcripts recovered in RNA-seq libraries of density-resolved total RNA from SIP gradients. Abundances are given relative to the total number of C23O transcripts in a given library. Only groups averaging >0.5 % of C23O transcripts in unamplified samples are shown. Isotope enrichment for taxa was calculated via EFs.

	Unamplified				Amplified				
	Unlabeled		Labeled		Unlabeled		Labeled		
	Light	Heavy	Light	Heavy	Light	Heavy	Light	Heavy	Average Abundance
Bacteria	100.0	100.0	100.0	100.0	100.0	100.0	100.0	100.0	100.0
Proteobacteria	99.4	98.3	94.7	98.4	97.2	97.8	97.6	98.0	97.7
Betaproteobacteria	11.2	17.4	15.1	13.2	11.3	13.0	19.5	17.8	14.8
Burkholderiales	1.1	0.9	1.3	1.1	2.1	2.5	2.4	3.0	1.8
Burkholderiaceae	0.0	0.0	0.0	0.0	0.0	0.3	0.8	0.0	0.1
Comamonadaceae	0.6	0.9	0.7	0.0	0.7	0.0	0.0	1.5	0.5
Hydrogenophaga	0.6	0.9	0.0	0.0	0.4	0.0	0.0	1.0	0.3
T4	0.6	0.9	0.0	0.0	0.0	0.0	0.0	1.0	0.3
Methylibium	0.6	0.0	0.0	0.0	0.0	0.0	0.0	0.0	0.1
Xenophilus	0.0	0.0	0.7	1.1	0.4	0.0	0.0	0.5	0.3
Xenophilus azovorans	0.0	0.0	0.7	1.1	0.4	0.0	0.0	0.5	0.3
Rhodocyclales	3.4	12.2	7.2	7.1	5.7	6.3	13.0	7.6	7.8
Rhodocyclaceae	3.4	12.2	7.2	7.1	5.7	6.3	13.0	7.6	7.8
Azovibrio	0.6	1.7	2.6	1.6	1.8	1.0	3.3	1.5	1.8
Azovibrio restrictus	0.6	1.7	2.6	1.6	1.8	1.0	3.3	1.5	1.8
unclassified Rhodocyclaceae	0.0	0.9	1.3	0.0	0.7	1.3	4.9	0.5	1.2
Rhodocyclaceae strain PG1-Ca6	0.0	0.9	1.3	0.0	0.7	1.3	4.9	0.5	1.2
Zoogloea	1.7	7.0	2.0	2.2	1.4	2.2	2.4	0.5	2.4
Zoogloea oleivorans	1.7	7.0	2.0	2.2	1.4	2.2	2.4	0.5	2.4
Methyloversatilis	0.0	0.0	0.0	0.5	0.0	0.0	0.0	0.0	0.1
Methyloversatilis universalis	0.0	0.0	0.0	0.5	0.0	0.0	0.0	0.0	0.1
Thauera	0.6	0.0	0.0	0.0	0.4	0.6	0.0	1.0	0.3
Gammaproteobacteria	7.3	13.0	6.6	10.4	9.2	5.4	15.4	10.2	9.7
Pseudomonadales	7.3	11.3	3.3	10.4	8.9	3.2	7.3	8.1	7.5
Pseudomonadaceae	5.0	11.3	3.3	7.1	6.4	3.2	7.3	8.1	6.5
Pseudomonas	5.0	11.3	3.3	7.1	0.4	3.2	7.3	7.1	5.6
Pseudomonas taeanensis	5.0	0.9	0.0	7.1	0.4	0.0	0.0	0.0	1.7
Xanthomonadales	2.2	0.9	3.3	3.3	2.1	2.2	7.3	1.5	2.9
Xanthomonadaceae	2.2	0.9	3.3	3.3	2.1	2.2	7.3	1.5	2.9
Pseudoxanthomonas	2.2	0.9	3.3	3.3	2.1	2.2	7.3	1.5	2.9
Pseudoxanthomonas spadix	2.2	0.9	3.3	3.3	2.1	2.2	7.3	1.5	2.9
Epsilonproteobacteria	0.0	0.0	0.0	0.0	0.4	0.3	1.6	0.0	0.3
Campylobacterales	0.0	0.0	0.0	0.0	0.4	0.3	1.6	0.0	0.3
Campylobacteraceae	0.0	0.0	0.0	0.0	0.4	0.3	1.6	0.0	0.3
Arcobacter	0.0	0.0	0.0	0.0	0.4	0.3	1.6	0.0	0.3

Table S4

Proportion of reads identified as non-rRNA and as mRNA in selected references.

Reference	Percent total reads non-rRNA	Percent total reads identified as mRNA	Database	Source
Radax et al. (2012)	8 %	1.5 %	NCBI-nr	Table 1
Fortunato and Huber (2016)	1.5 - 75.5* %	0.04 – 2.5 %	KEGG Orthology	Calculated from Table S1
Schwab et al. (2014)	1.7 – 7.7 %	0.6 – 2.2 %	SEED	Calculated from Table S1

* The high percent of reads not identified as rRNA in some samples is not discussed in Fortunato and Huber (2016), but may be due to an abundance of uncultivated lineages at these sites and/or their use of an older Silva release (release 111). The percent of total reads matching mRNA sequences in the KEGG database remains < 2.5 % despite the high percent of reads considered non-rRNA.

Supplementary Figures

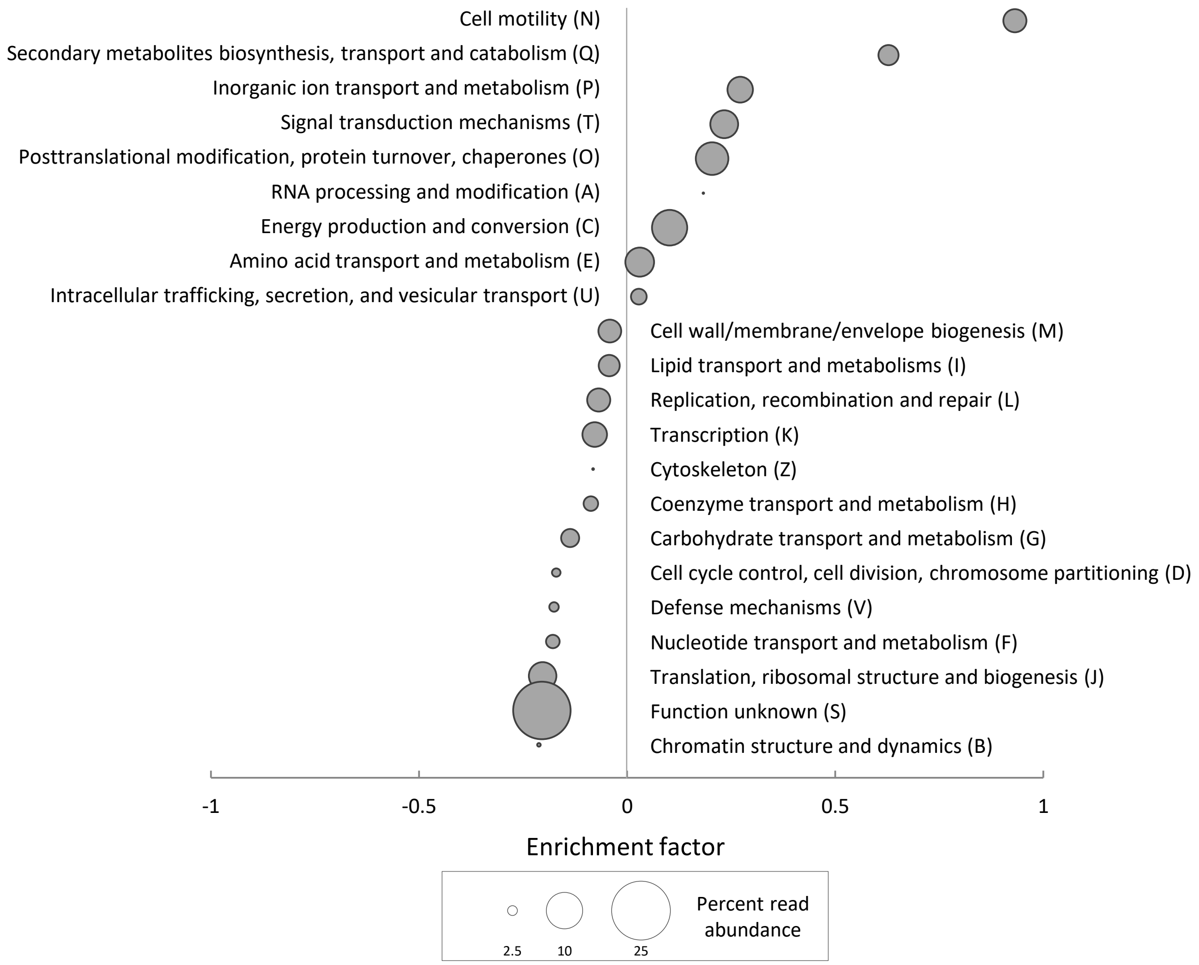

Figure S1

^{13}C-labelled mRNA transcripts identified by RNA-SIP in toluene-degrading microcosms. Rankings of mRNA enrichment factors (EFs) are resolved at the level of COG categories. EFs are shown in combination with relative read abundances averaged across all eight RNA-seq libraries. Individual transcripts shown are those with ≥ 20 total reads.

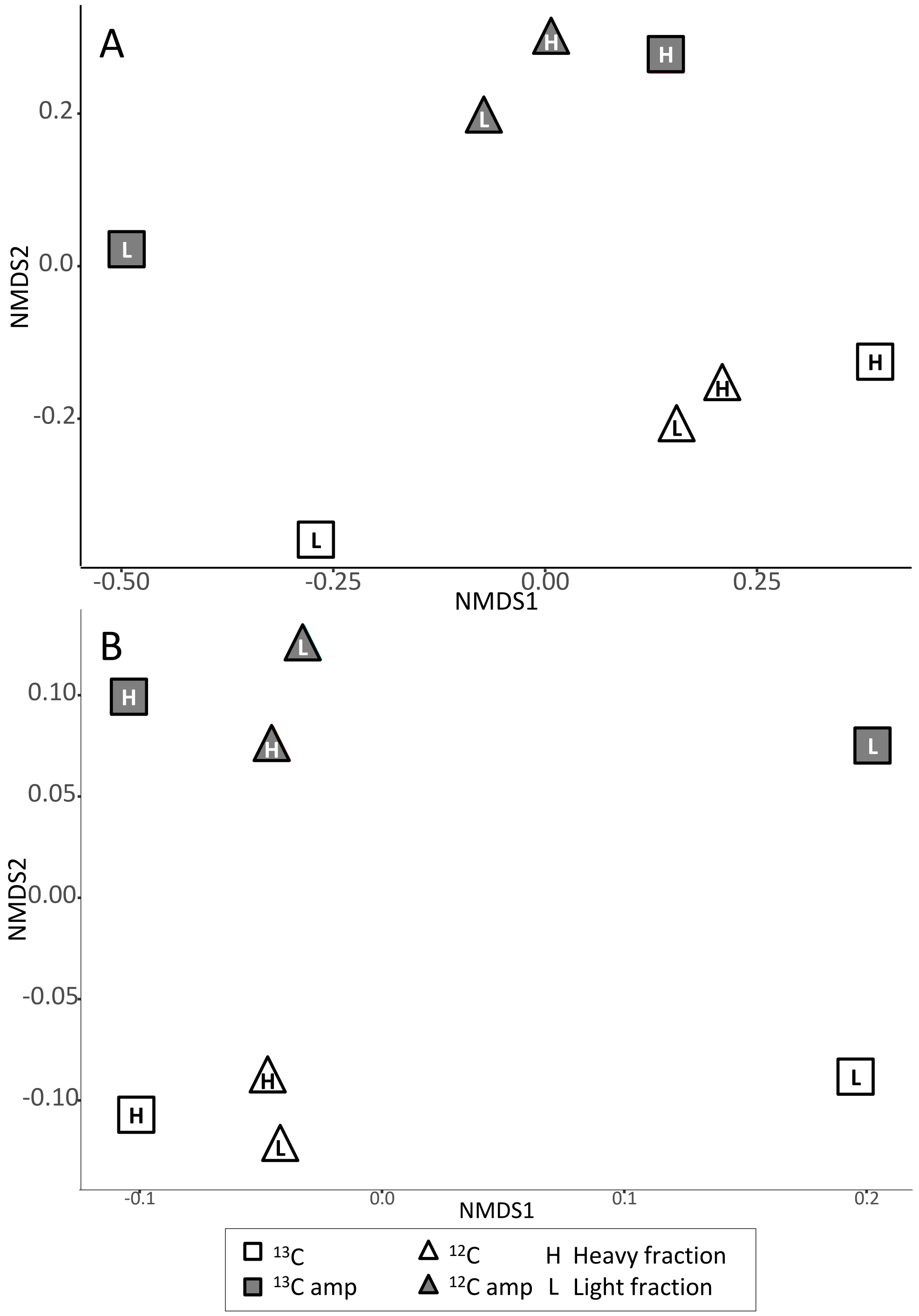

Figure S2

(A) NMDS ordination of profiles of SSU rRNA reads as identified in unamplified and amplified RNA-seq libraries of RNA-SIP fractions. (B) (B) NMDS ordination of profiles of functional transcript reads as identified in unamplified and amplified RNA-seq libraries of RNA-SIP fractions using COG.

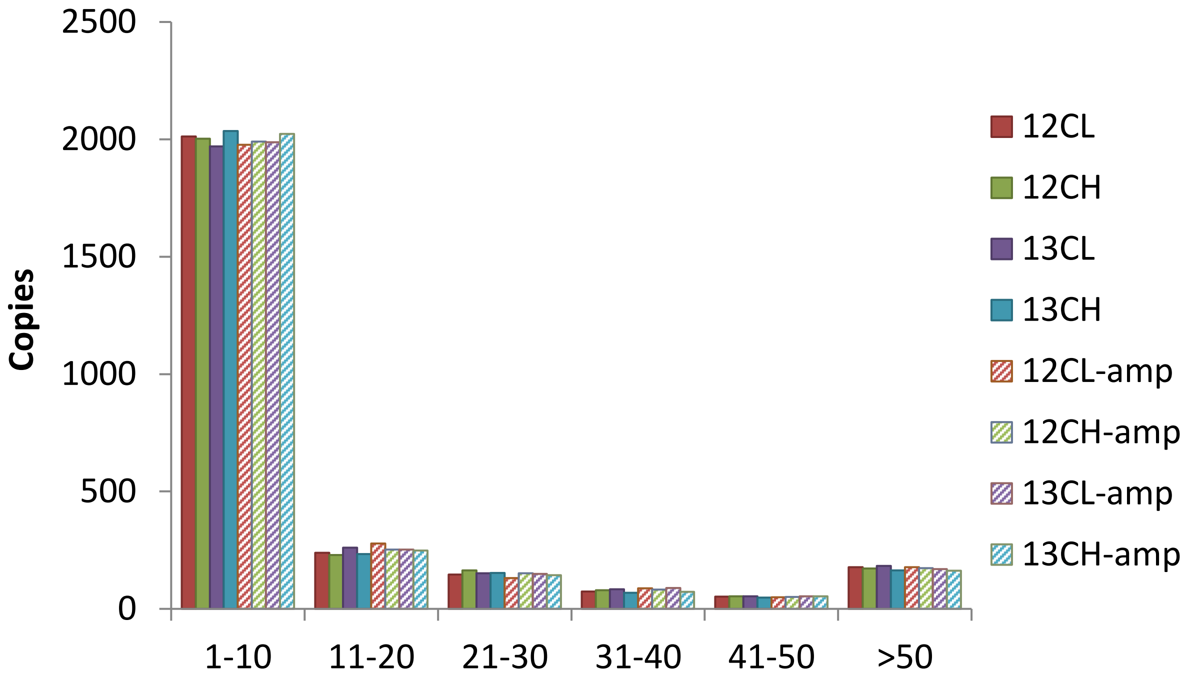

Figure S3

Abundance distribution of the quantitative assignment of sequencing reads to specific functional transcripts in unamplified and amplified RNA-seq libraries of RNA-SIP fractions using KEGG.

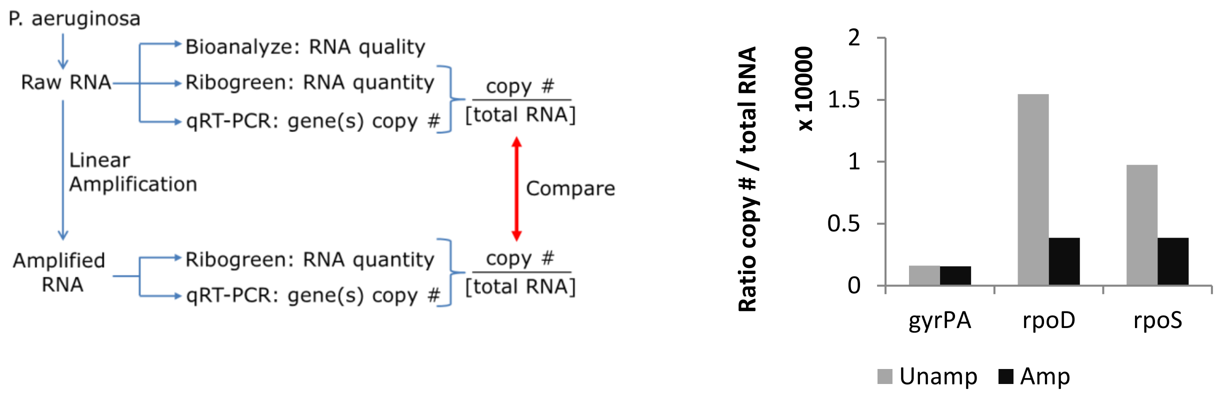

Figure S4

Schematic workflow and RT-qPCR results of expressed housekeeping genes in unamplified and amplified total RNA of a pure culture of *P. aeruginosa*.
